# Trends and clinico-epidemiological features of human rabies cases in Bangladesh 2006–2018

**DOI:** 10.1038/s41598-020-59109-w

**Published:** 2020-02-12

**Authors:** Sumon Ghosh, Md. Sohel Rana, Md. Kamrul Islam, Sukanta Chowdhury, Najmul Haider, Mohammad Abdullah Heel Kafi, Sayed Mohammed Ullah, Md. Rashed Ali Shah, Afsana Akter Jahan, Hasan Sayedul Mursalin, Aung Swi Prue Marma, S. M. Emran Ali, Shohrab Hossain, Rajub Bhowmik, Nitish C. Debnath, Abul Khair Mohammad Shamsuzzaman, Be-Nazir Ahmed, Umme Ruman Siddiqi, Sanya Tahmina Jhora

**Affiliations:** 1 Disease Control Unit, Communicable Disease Control, Directorate General of Health Services, Ministry of Health and Family Welfare, Dhaka, Bangladesh; 2 International Centre for Diarrhoeal Disease Research, Bangladesh (icddr,b), Dhaka, Bangladesh; 3 Technical University of Denmark, Section for Epidemiology, National Veterinary Institutes, Copenhagen, Denmark; 4 Faculty of Veterinary and Animal Sciences, Gono University, Savar, Dhaka, Bangladesh; 5 Infectious Disease Hospital, Directorate General of Health Services, Ministry of Health and Family Welfare, Dhaka, Bangladesh; 6 Tongi Municipality, Tongi, Bangladesh; 7 John Jay College of the City University of New York, 445 W 59th St-10019, New York, USA; 8 Food and Agriculture Organization of the United Nation, Dhaka, Bangladesh; 9 Department of Livestock Services, Ministry of Fisheries and Livestock, Dhaka, Bangladesh; 10 Department of Pathobiology and Population Sciences, Royal Veterinary College, University of London, London, UK

**Keywords:** Viral infection, Viral infection, Epidemiology, Epidemiology

## Abstract

Vaccinating dogs against rabies is an effective means of reducing human rabies. We subjected 1327 clinically diagnosed human rabies death and mass dog vaccination (MDV) data during 2006–2018 to quantify the impacts of MDV on human rabies incidence in Bangladesh and a subset of rabies death data (422) for clinico-epidemiological analysis. A positive and increasing trend of MDV (p = 0.01 and tau = 0.71) and a negative and declining trend (p < 0.001 and tau = −0.88) of human rabies cases (Correlation coefficient: −0.82) have been observed. Among 422 deaths, the majority (78%) of the victims sought treatment from traditional healers, and 12% received post-exposure prophylaxis (PEP). The mean incubation period of rabies in cases with exposure sites on the head & neck (35 days) was shorter than the upper limb (mean = 64 days, p = 0.02) and lower limb (mean = 89 days, p < 0.01). MDV has been found to be effective for reducing human rabies cases in Bangladesh. Creating awareness among the animal bite victims to stop reliance on traditional healers rather seeking PEP, addressing the role of traditional healers through awareness education programme with respect to the treatment of dog bites, ensuring availability of PEP, and continuing to scale up MDV may help to prevent human rabies deaths.

## Introduction

Rabies is a zoonotic viral disease responsible for the death of approximately 59,000 people worldwide with more than 3.7 million disability-adjusted life years lost annually^1^. Due to acute progressive encephalitis, rabies is almost always fatal once clinical signs appear. The disease occurs predominantly in impoverished communities, in both rural and urban areas, and has been recognized for over 4000 years^2,3^. Rabies is present across all continents, except Antarctica with more than 95% of human fatalities happening in the areas of Asia and Africa, and approximately 40% of cases in the population are aged below 15 years. Although all warm-blooded animals are susceptible to rabies, domestic dogs are the main cause of rabies virus transmission to humans in up to 99% of cases in rabies-endemic regions^4^. Most cases of rabies are caused by the bite of an infected dog. The effect of rabies virus (RABV) exposure depends on a number of factors, including the gravity of the wound, the anatomical site of the bite on the body, the viral quantity and variant (genotype) inoculated into the wound(s) and the timeliness of post-exposure prophylaxis (PEP)^3^. The clinical manifestation of human rabies can appear in any of two forms: the widely perceived furious (classical or encephalitic) form or the paralytic (or dumb) form^5,6^. Each case of rabies has its own diverse clinical characteristics, which may be related to different viral tropisms and neural sites, courses of neural spread, variable immune responses and/or potentially different pathological mechanisms^7–9^.

Globally, the WHO and the World Organisation for Animal Health, in collaboration with the UN Food and Agriculture Organization and Global Alliance for Rabies Control, also known as “United Against Rabies,” called for the elimination of dog-mediated human rabies by 2030 and are providing/have agreed to provide technical supports towards the elimination of dog mediated human rabies^10,11^.

Controlling rabies in dogs can significantly reduce human exposure^12^. Vaccinating domestic dogs against rabies is an effective means of controlling rabies in dogs by disrupting the transmission of RABV between dogs. If coverage of 70% can be achieved in annual pulse vaccination campaigns, the rabies incidence in dogs is likely to be dramatically reduced, and if this coverage is maintained over periods of several years, regional elimination is possible^3,13^. This rabies elimination approach has been demonstrated in various settings in Africa, Asia, Europe, and the Americas^3,12,13^.

Rabies remains endemic in Bangladesh and has high public health importance^14^. However, the number of deaths from human rabies has decreased by approximately 50 per cent in recent years, resulting from a combined government effort involving advocacy, communication and social mobilization (ACSM), modern treatment for animal bites, mass dog vaccination (MDV) and dog population management (DPM)^15–17^. While ACSM and animal bite management have been continuing, MDV was first piloted in Bangladesh in November 2011, covering a small municipality, namely, Cox’s Bazar^18^. Upon successful piloting, more comprehensive campaigns of MDV were scaled up throughout the country.

An evaluation of MDV against rabies and other factors affecting the advancement of the continuous rabies control programme is essential for rolling out fundamental improvements to future programme implementation^19^. In addition, knowledge regarding the trends and clinico-epidemiological features of human rabies is crucial to direct further research and to implement disease control measures^9^. Therefore, this study was aimed to understand the impacts of MDV and to describe the trends and clinico-epidemiological features of human rabies cases in Bangladesh.

## Methods

### Data source

We obtained data from the record books of patients with animal bites and rabies cases treated at the National Rabies Prevention and Control Center (NRPCC) of the Infectious Disease Hospital (IDH) in Dhaka, Bangladesh, from January 2006 through December 2018. IDH (NRPCC) is the primary referral centre for animal bites and rabies patients in Bangladesh, and hence, most cases of animal bites from different parts of the country come up here for free vaccination and treatment^20^. Other than NRPCC, there are 66 public District Rabies Prevention and Control Centers (DRPCCs) with at least one centre in each of the 64 districts that provide a free anti-rabies vaccine (ARV) and treatment for victims of dog bites^16,21^. However, all the rabies suspect cases have to be referred to IDH (NRPCC) from the DRPCCs and different areas of the country for further management and data recording. All rabies cases were diagnosed clinically and not by the laboratory test. This situation was inevitable due to the socio-cultural practices that the relatives did not permit brain tissue sampling for confirmation and poor laboratory diagnostic facilities in Bangladesh.

Data included demographic information regarding the patient’s origin, age, and sex. Clinical data contained but was not limited to the estimated date of animal exposure, bite site, type of exposure, incubation period (the time between the exposure and the manifestation of signs and symptoms), the presence of various prodromal clinical signs and signs of dysfunction in the central and autonomic nervous system, health-seeking behaviour following animal exposure, and PEP utilization by the animal bite victims.

The Communicable Disease Control Division (CDC) of the Directorate General of Health Services (DGHS) of Bangladesh was coordinating the MDV programme under the National Rabies Elimination Programme. They also provided dog rabies vaccines for conducting MDV. Along with DGHS, the Department of Livestock Services, the Local Government Division, Education sectors, and Non-Government Organizations or development organizations, all contributed in different ways to the MDV programme in Bangladesh. For data regarding MDV, we used the MDV database of the CDC, DGHS from November 2011 through December 2018. The dog population was estimated during the MDV campaign using the ‘Capture-mark-recapture’ method that consists of temporarily marking dogs, e.g., with a dye or distinctive collars^22,23^. We divided the dog population into three ownership types in this paper: (1) ‘stray dogs’ to be ownerless, seen in public areas and are not restricted to the house or property, therefore, have no health care and must forage for their own food, (2) ‘community dogs’, dogs without a particular owner that roamed in a particular community but lived on food mainly provided by the people of that community, and (3) ‘pet dogs’ that are fed regularly and have access to health care through their owners. The surveyors of the post MDV survey, have identified the dogs in this way during the campaign.

### Data analysis

The MDV campaigns are usually conducted annually for particular areas and the duration of immunity persists for 1–3 years^24^. In this study, we considered that immunity against rabies in vaccinated dogs would persist for two years. The programme was implemented in different phases to complete vaccinating all the dogs of a district/area in a three year period. In the first phase, (Year 1) some areas/sub-districts of a district (typically a district of Bangladesh consists of 3–14 sub-districts) were selected and dogs of those areas/sub-districts were vaccinated. In the next phase (Year 2), the remaining areas/sub-districts of the same district were selected, and dogs were vaccinated. The areas/sub-districts selected each year was different until year 3. The number of dogs vaccinated in Year 1 and Year 2 were added together to identify the number of dogs vaccinated until the second year. We considered the possibility of multiple vaccinations of the same dog. While we acknowledge the limited potential for movement to a new area/sub-district, we ignored this minimal chance due to the lack of data to consider this scenario. We performed the Mann-Kendall trend test to identify the trend of human rabies cases over the period of 2006–2018, as well as the number of dogs vaccinated over the period of 2011 to 2018. Additionally, we conducted Sen’s slope test to identify the changes in human rabies cases annually^25^. Finally, we executed a correlation coefficient test to identify the relationship between human rabies cases (2006–2018) and the number of dogs being vaccinated against rabies (2011–2018). We hypothesized that bites closer to the brain would have a shorter incubation period and compared the incubation period of rabies in persons exposed to the head and neck with persons exposed to other parts of the body and thus tested a null hypothesis for this. We performed Student’s t-test to identify the difference between the incubation period of rabies in cases with exposure sites on the head & neck and in other organs and reported the p-value.

We generated a map to show the regional variation of rabies deaths and death rates (cases per 100,000 populations) in Bangladesh. District wise death rates are standardized to 2011 population estimates^26^. We created a heat map to demonstrate year-wise MDV and its coverage in different districts of Bangladesh and a box plot to show the incubation period according to the site of the exposure. Using descriptive statistics, we analyzed different clinical and epidemiological characteristics of rabies cases reported in the IDH, Dhaka, Bangladesh, from 2011 to 2015.

## Results

**Figure 1:**
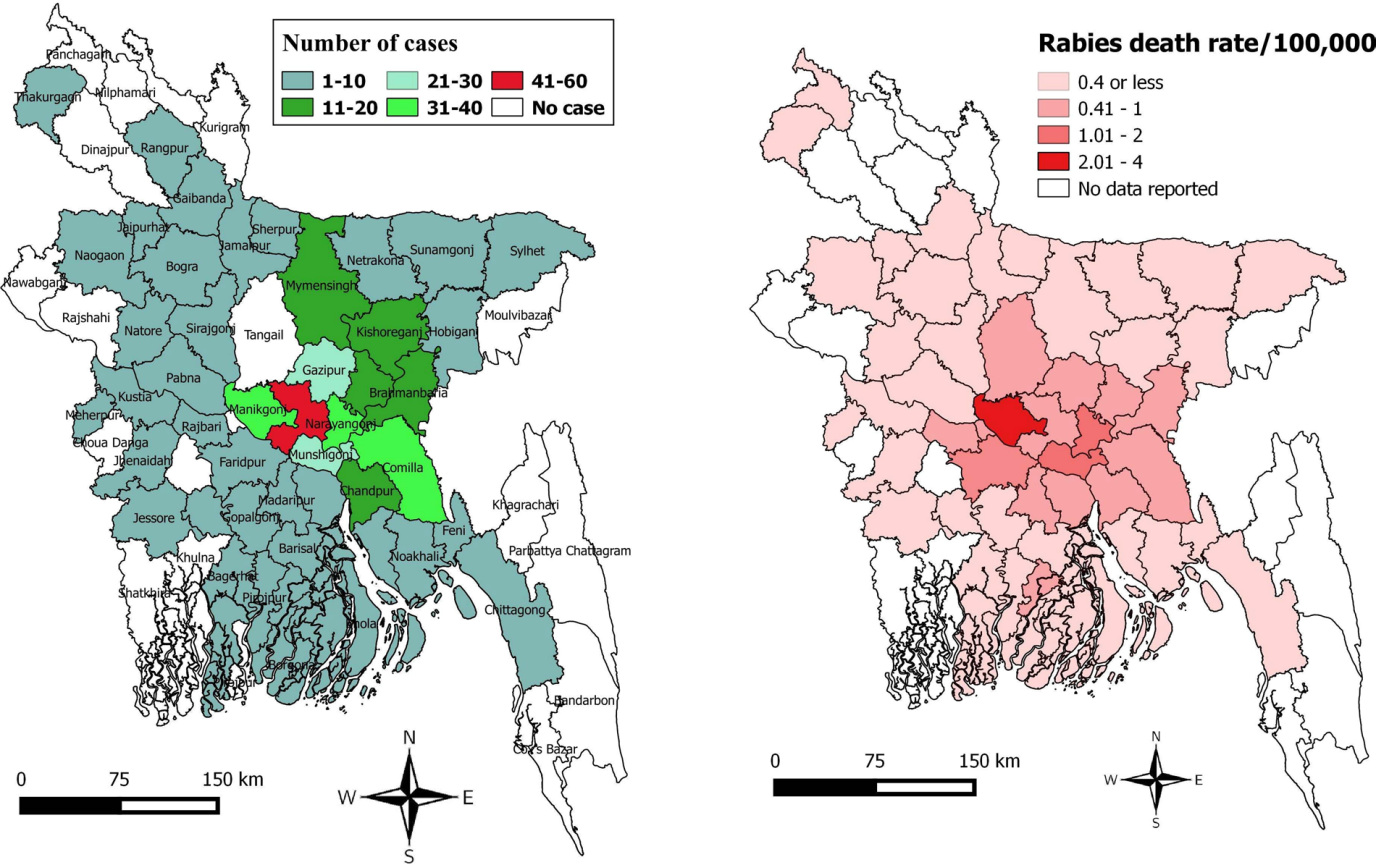
Regional variation of rabies deaths and death rates in Bangladesh. (**a**) Distribution of human rabies cases from different districts of Bangladesh reported at the National Rabies Prevention and Control Centers (NRPCC) of the Infectious Disease Hospital (IDH), Dhaka, Bangladesh, 2011–2015 (**b**) Regional variation of rabies death rates (cases per 100,000 populations) in Bangladesh.

We analysed 1327 human rabies death data reported at the IDH, Bangladesh, from 2006 to 2018 to explore the trends of human rabies over time. A subset of those rabies case data (422 cases from 2011 to 2015) was reviewed for the clinico-epidemiological study. The highest number of cases was observed in the districts in the middle of the country (Fig. 1).

### Socio-demographic characteristics of the deceased rabies victims

There were a high number of rabies deaths in males when compared to females (296 vs. 126). Almost half of the deceased rabies victims (n = 201, 47%) were under the age of 15 with a median age of 18.5 years (Figure S1). A total of 346 (82%) rabies victims came from rural areas, and 88% (370) of the cases died at home (Table 1).

**Table 1:** Demographic and socioeconomic characteristics of deceased rabies victims reported at the National Rabies Prevention and Control Centers (NRPCC) of the Infectious Disease Hospital (IDH), Dhaka, Bangladesh, 2011–2015.

Variables/categories	N = 422 n (%)
Gender-male	296 (70)
Mean age (in years, range)	26.5 (2–90)
**Age (years)**	
<15	201 (47)
15–30	63 (15)
30–45	67 (16)
45–60	67 (16)
60–90	24 (6)
**Occupation**	
Dependent (including children)	282 (67)
Farming	63 (15)
Business	48 (11)
Day labour	29 (7)
**Residence**	
Rural	346 (82)
Urban	76 (18)
**Dog ownership**	10 (2)
**Place of death**	
In a health facility/hospital	52 (12)
At home/other location	370 (88)

### Characteristics of animals to which the deceased rabies victims were exposed

Dogs comprised the majority of exposure sources (n = 380, 90%) followed by cats (n = 24, 6%), jackals (n = 12, 3%) and mongooses (n = 6, 1%). Stray dogs contributed the majority of exposures (n = 390, 93%) while wild, pet and community animals made up 4% (n = 18), 2% (n = 10) and 1% (n = 4) of cases, respectively. Seventy-four per cent (n = 312) of the animal bite cases took place without any provocation by the victims. In almost all cases, the vaccination status of the attacking animals was unknown (n = 412, 98%) (Table S1).

### Characteristics of animal exposure to the deceased victims

Animal bites accounted for most of the exposures (n = 399, 95%), whereas the remaining was scratch either by teeth (n = 13, 3%) or by claws (n = 10, 2%), and 56% of cases were attributed to a single bite (n = 223). The anatomical position of the bites involved mainly the lower limbs (n = 320, 76%) with the majority of the bites being category III (n = 399, 95%) (WHO category of exposure) (Table 2).

**Table 2:** Characteristics of animal exposure to the deceased rabies victims reported at the National Rabies Prevention and Control Centers (NRPCC) of the Infectious Disease Hospital (IDH), Dhaka, Bangladesh, 2011–2015.

Variables/categories	N = 422 n(%)
**Nature of the exposure**	
Bite	399 (95)
Scratch by teeth	13 (3)
Scratch by claws	10 (2)
**Anatomical site of exposure**	
Trunk	21 (5)
Head & neck	42 (10)
Upper limb	39 (9)
Lower limb	320 (76)
**Categories of contact**	
Category II	23 (5)
Category III	399 (95)
**Number of bites (N = 399)**	
Single	223 (56)
Multiple	176 (44)

### Heath seeking behaviour following animal exposures

Seventy-eight per cent (n = 327) of the victims sought treatment from traditional healers (faith healers generally entrusted with minor medical illnesses). Only 12% (n = 51) received PEP, and among the patients who received PEP, 84% (n = 43) received an intradermal rabies vaccine (IDRV) [commercial tissue culture vaccines, Rabix-VC® (Incepta Pharmaceuticals, Ltd., Bangladesh)] but did not receive the complete vaccination series [WHO approved Thai Red Cross (TRC) ID regimen]. Only 6 (12%) patients received the complete vaccination series; however, they experienced an average of 5 days (range: 4–7 days) of treatment delay in receiving vaccines with no rabies immunoglobulin (RIG) (Table 3).

**Table 3:** Heath seeking behaviour following animal exposures among the rabies victims reported at the National Rabies Prevention and Control Centers (NRPCC) of the Infectious Disease Hospital (IDH), Dhaka, Bangladesh, 2011–2015.

Variables/categories	N = 422 n (%)
**Treatment-seeking behaviours**	
Received traditional treatment	327 (78)
Rabies PEP received	51 (12)
Consult with local doctors	38 (9)
No measures taken	6 (1)
**History of proper wound washing**	
No	162 (39)
Yes	127 (30)
Unknown	133 (31)
**Types of vaccine received, (N = 51)**^**#**^	
Intradermal rabies vaccine (IDRV)	43 (84)
IDRV + rabies immunoglobulin (RIG)	2 (4)
Nerve tissue vaccine (NTV)	6 (12)
**Vaccination facility, (N = 51)**^**#**^	
District Rabies Prevention and Control Centre (DRPCCs)	16 (31)
Infectious Disease Hospital (IDH)	1 (2)
Institute of Public Health (IPH)	10 (20)
Municipality	3 (6)
Pharmacy	18 (35)
Sub-district hospital (Thana Health Complex)	3 (6)
**Course of vaccination, (N = 51)**^**#**^	
Complete	6 (12)
Incomplete	45 (88)

#Described the subset of the data, i,e, 51 patients that received rabies PEP after animal exposure.

**Figure 2:**
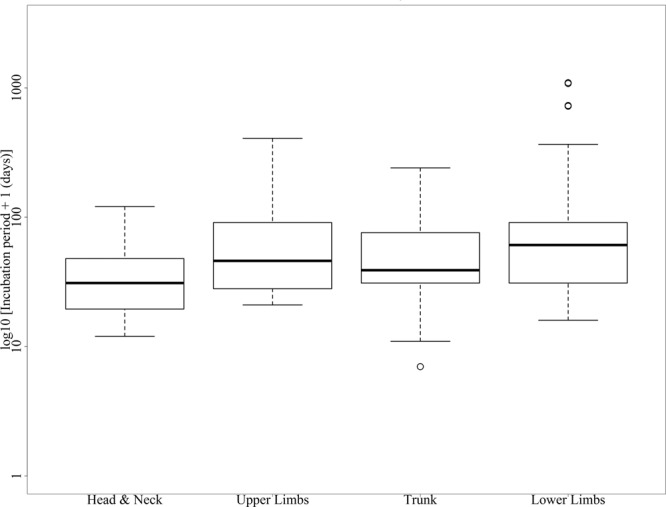
The estimated incubation period (in days)* of rabies per anatomical location of animal bite wounds reported at the National Rabies Prevention and Control Centers (NRPCC) of the Infectious Disease Hospital (IDH), Dhaka, Bangladesh, 2011–2015. The bottom and top of the box indicate the first and third quartiles, respectively; the band inside the box is the median. The dots outside the box are individual outliers *Period from the time of exposure to the time of manifest signs and symptoms.

The incubation period of rabies ranged from 6 days (lowest incubation period) to 1095 days (highest incubation period), with a median of 52 days (Fig. 2). The median incubation period of rabies was 30 days (range: 11–120 days) in cases with exposure sites on the head & neck; 38 days (range: 6–240 days) for the trunk; 45 days (range: 20–407 days) for the upper limbs; and 60 days (range: 15–1095 days) for the lower limbs. The mean incubation period of rabies in cases with exposure sites on the head and neck (35 days) was shorter than those for the upper limb (mean = 64 days, p = 0.02), lower limb (mean = 89 days, p < 0.01) and trunk (mean = 58 days, p = 0.053).

### Rabies cases, according to the type of clinical manifestation

The clinical symptoms were recorded during the patient’s admission (Table 4). Almost all patients (n = 409, 97%) demonstrated hydrophobia upon admission. Aerophobia occurred in 84% (n = 353) of the patients, elicited using the fan test. Photophobia was noted in 10% (n = 42) of patients.

**Table 4:** Rabies cases according to the type of clinical manifestation reported at the National Rabies Prevention and Control Centers (NRPCC) of the Infectious Disease Hospital (IDH), Dhaka, Bangladesh, 2011–2015.

Variables/categories	N = 422 n(%)
**Clinical manifestation**	
Hydrophobia	409 (97)
Aerophobia	353 (84)
Fever	86 (20)
Photophobia	42 (10)
Hypersalivation	29 (7)
Restlessness	27(1.4)
Vomiting/nausea	24 (6)
Itchiness of the bite site	24 (6)
Lethargy	14 (3)
Anxiety	13 (3)
Unusual sensation	12 (3)
Dysphagia	10 (2)
Respiratory distress	4 (<1)
Convulsion	4 (<1)

### MDV, PEP, and trends of human rabies

**Figure 3:**
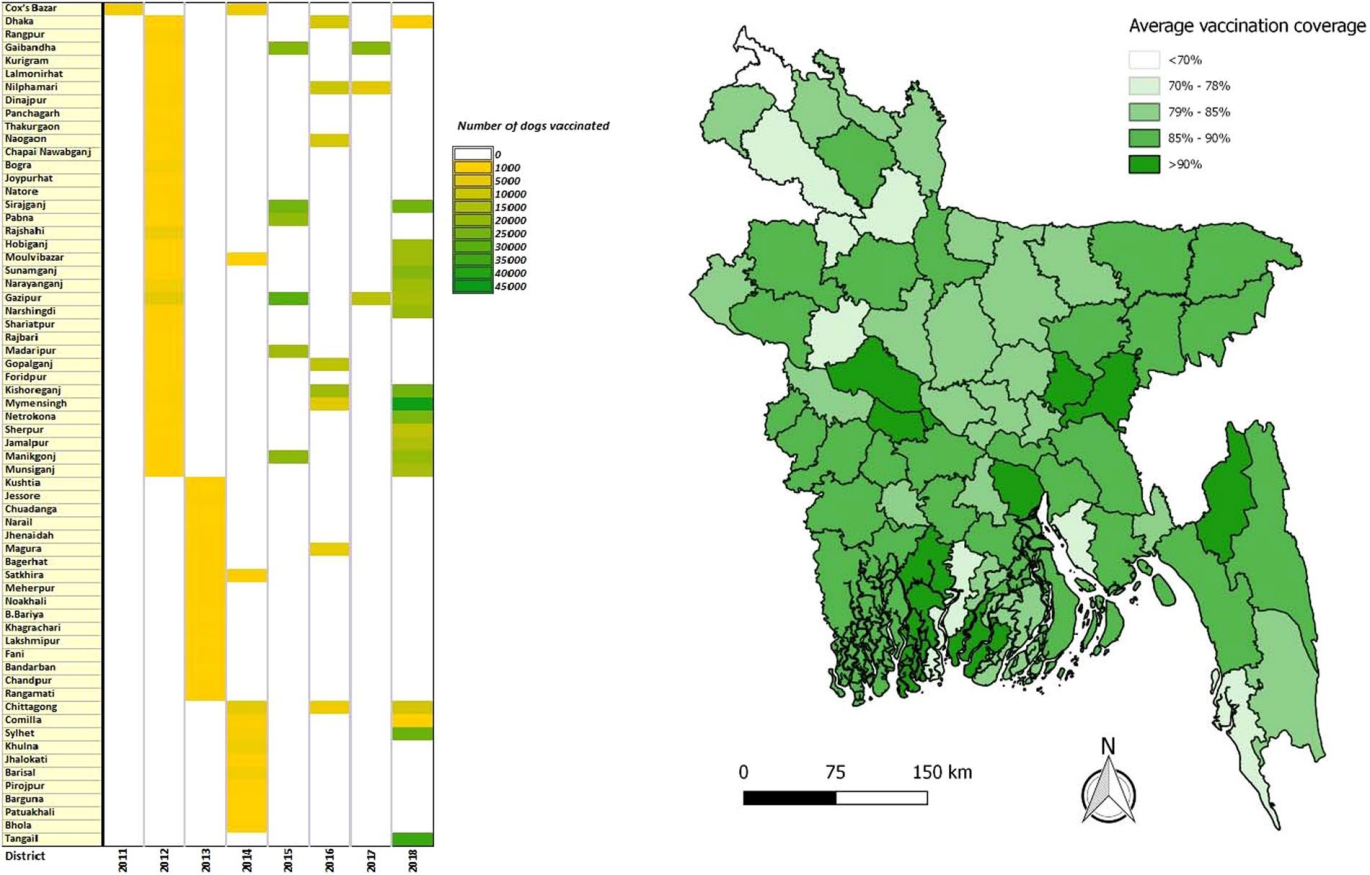
Scaling up mass dog vaccination (MDV) in different districts of Bangladesh with vaccination coverage, 2011–2018. (**a**) Year-wise number of dogs vaccinated in different districts of Bangladesh. (**b**) Average vaccination coverage in different districts of Bangladesh.

**Figure 4:**
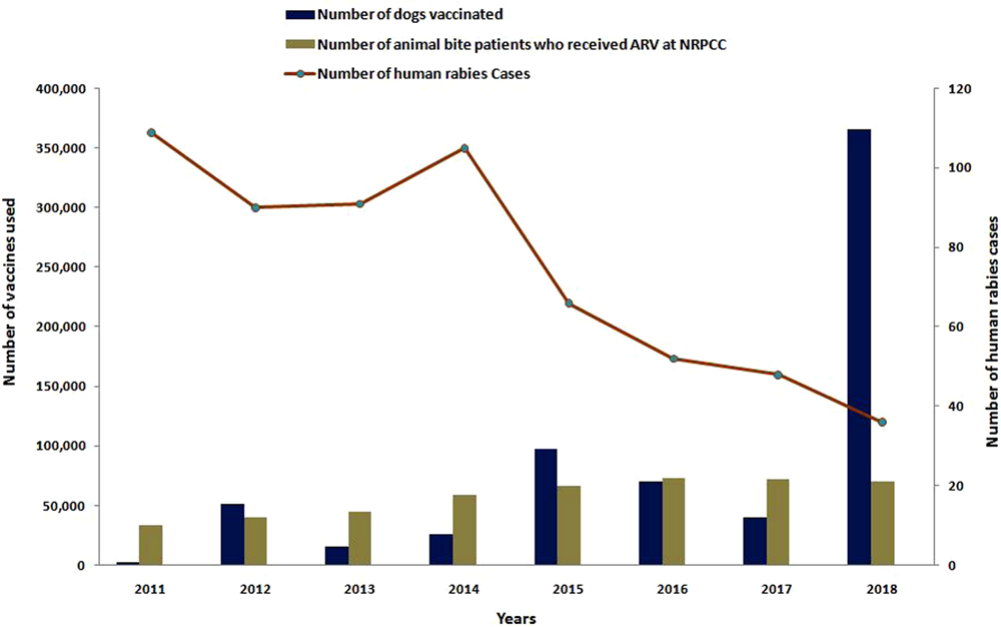
Impact of Mass Dog Vaccination (MDV) and Post-Exposure Prophylaxis (PEP) on human rabies incidence in Bangladesh, 2006–2018.

We analysed 8 years (2011–2018) of MDV data maintained at the DGHS of Bangladesh. During this period, MDV was scaled up for at least one round in all 64 district municipalities of the country, and of these, all areas of 23 selected districts were covered with MDV. Three rounds of MDV were scaled up in three areas, namely, Cox’s Bazar district municipality, Satkhira Sadar sub-district, and Sreepur sub-district (Figure S2) of Bangladesh. Under the MDV campaign, out of an estimated 871,765 dogs, an estimated 81% (95% CI: 80.88–81.04) have been vaccinated. From 2011 onward, an upward trend of MDV was found, and the highest number of dogs was vaccinated in 2018 (n = 365,316) (Fig. 3) (Tables S2, S3). From 2011 to 2015, a total of 942,378 animal bite cases managed at NRPCC and DRPCCs of Bangladesh with an average of 188475 cases managed each year (Table S4). We also found an increasing trend of ARV utilization and dog vaccination and a decreasing trend of human rabies cases in Bangladesh from 2011–2018 (Fig. 4) (Figure S3).

We identified a trend of declining human rabies cases in Bangladesh from 2006–2018 (p < 0.001 and tau = −0.88). Using Sen’s slope test, we found that over the 13-year period, the slope was 11.31 cases (95% CI: −9.00 to −14.00) (p < 0.001). On the other hand, we found a positive trend of dog population immunization from 2011–2018 (p = 0.01 and tau = 0.71). In Sen’s slope test, for dog population vaccination over a period of 8 years, the slope was 31,162 (95% CI: 11,192–67,634) per year (Fig. 4). The correlation coefficient between the number of human rabies cases and the number of dogs being vaccinated against rabies was −0.82 during the period of 2011 to 2018.

## Discussion

Our study has identified several important clinico-epidemiological characteristics of human rabies cases and provides scientific information on the benefits of dog rabies vaccination on human rabies incidence that could be helpful for the structuring of suitable and successful dog mediated rabies control strategies in Bangladesh. In some instances, public health agencies around the developing world make substantial investments for identifying successful interventions to solve emerging public health problems but hardly ever pay enough attention to the known interventions. Our data showed that complex public health problems could be resolved with known interventions like MDV. This is a good example of how zoonotic diseases should be controlled with a true One Health approach. In addition to MDV, there are few other factors, such as raising awareness and access to PEP by the animal bite victims, that may explain changes in the detection of human rabies cases in Bangladesh. A key aim of the national rabies elimination plan of the government of Bangladesh (GOB) is dedicated to building awareness and focusing on the importance of prevention. To that end, the GOB has marked several events with ACSM like educational programmes in primary schools across the country and through a series of press conferences, seminars, and rallies^15^. Along with MDV, there are other two proven, effective interventions, namely, awareness and PEP, that have eliminated dog-mediated human rabies in Western Europe, North America, Japan, South Korea, and parts of Latin America, Africa and Asia^27^. During the eight-year period, we found that a total of 705,839 dogs have been vaccinated in Bangladesh, with >70% coverage achieved in almost all the areas where MDVs have scaled up. In view of the recommendations of the WHO, it is estimated that a country needs to vaccinate 70% of its total dog population for seven years in order to eliminate rabies from dogs^28^.

We identified a downward trend of human rabies cases in Bangladesh from 2006–2018 and an upward trend of dog immunization from 2011–2018. Over the 13-year period, annual cases reduced at a rate of approximately 12 per year, and over a period of 8 years, the number of vaccinated dogs increased at a rate of 3200 dogs per year. The time series for human rabies cases was available over a longer period (2006–2018) than that for MDV data (2011–2018). The trend of human rabies cases changed quite rapidly after MDV was introduced in Bangladesh in 2011. The combined impact of a mass awareness programme, PEP, and MDV has resulted in a consistent decrease in the annual incidence of rabies in Bangladesh^17^. Sri Lanka and Bhutan have also shown noticeable progress in their rabies elimination programme through awareness education and successful implementation of MDV^29,30^. In Bangladesh, all 64 districts now have at least one centre providing wound management and PEP to animal bite victims totalling more than 250,000 patients treated by trained nurses and physicians, incurring no out-of-pocket expenses to the patients. The expansion of MDV has reaffirmed how a multi-sectoral, One Health approach conjoining innovation, capacity-building and widespread implementation can lead to effective rabies elimination strategies^17^.

The epidemiological characteristics of human rabies cases found in this study were similar to those reported elsewhere in Bangladesh and neighbouring countries. Our study revealed that rabies victims were mostly males of less than 15 years of age, which is similar to some other studies in Bangladesh and neighbouring countries^14,16,31^. The bite locations were mostly on the patients’ lower limbs, with the majority of the bites being category III^4^. Similar results have been documented in other studies in developing countries^31,32^. However, bites on the head and neck were not infrequent (10%) in our study. In China^33^ and Tanzania^34^, bites on the head and neck region have made an important contribution to human rabies. Exposure of the upper body and limbs (head, neck, arm, or hand) to a rabid animal is likely to have more adverse consequences than the exposure of the lower parts of the body^31^. The median danger-of-death toll after exposure of the head, hand, trunk, and legs to a rabid animal was reported to be 45%, 28%, 5%, and 5%, respectively^31,35^. In our study, we found that 5% of the patients developed rabies due to scratches or abrasions without bleeding either by cats or by puppies; however, we were uncertain whether the patient’s attendants recalled the event correctly. Similar findings have also been documented in other studies in Bangladesh and India^20,36^. WHO recommends a rabies vaccine for category II bites that include minor scratches or abrasions without bleeding, and additional local RIG infiltration may be necessary^36^.

We found a difference in incubation times, depending on the sites of exposure. Similar findings have also documented in another study where the incubation period was shorter in cases with exposure sites on the head and neck than in cases with a wound on the trunk, upper or lower limbs^37^. The incubation period depends on a number of factors including the site and type of bite wound(s), virus inoculum and rapid treatment of wounds, viral strains, and host factors^9,38,39^. Our study identified three patients with unusual incubation time (two for 1095 and one 1080 days) which might be due to recall bias. In rabies endemic areas, there could have been recurrent exposures following the first bite. Some other studies have documented a much longer incubation time (27 years in the Philippines) than usual for rabies^9,40^. It may be difficult to diagnose rabies if the incubation period is longer, especially when the exposure history is missing^41^. We found that bites in the head and neck region seemed to have a shorter incubation time in contrast to that for bites in the lower extremity. Other studies have also demonstrated a similar phenomenon^9,42^.

Our study showed the manifestation of hydrophobia evoked upon admission in the vast majority of cases (97%). Hydrophobia can be seen in classic rabies in 50–80% of cases^43^. Despite the fact that there are no confirmatory laboratory diagnostic facilities for human rabies in Bangladesh, a history of an animal bites and evidence of hydrophobia along with the characteristics of a rapidly fatal encephalopathy, have enabled us to diagnose the cases as rabies. We also observed a high percentage of victims (84%) manifesting aerophobia, which is another hallmark feature of rabies. This finding is parallel to other studies conducted in Bangladesh^44^ and its’ neighbouring countries^45^.

Our analysis showed that the majority of the rabies victims primarily sought treatment from traditional healers following animal exposure, with no history of taking any PEP. Previous studies in Bangladesh revealed that a good number of animal bite victims first sought treatment from traditional healers instead of taking any modern PEP^16^. These sorts of health-seeking behaviour might be attributed to persevering myths and dogmas among the people of the community, poor socio-economic status, lack of proper education, lack of accessibility and affordability of PEP treatment in local government hospitals, distance to government hospitals from the victims’ residence, etc.^16^. The following measures should be taken to prevent the dog mediated human rabies: setting up awareness and education campaigns and implementing dog bite prevention strategies, especially targeting the children, encouraging and educating about responsible dog ownership and conceptualizing cultural differences that impact the societal role and value of dogs. The WHO recommends immediate wound washing along with vaccination following exposure to a suspected rabid animal, which would help to prevent nearly 100% of rabies deaths. However, in type III wounds, RIG must be required to be infiltrated in and around the wound(s)^24^.

PEP for rabies involves recurrent visits to the treatment centre by the bite victims to maintain a complete course within 28 days. Hence, emphasis must be placed on patient compliance to make the immunization appropriate^46,47^. Our study unveiled that among the patients who received PEP, only 12% followed the complete vaccination series (Thai Red Cross ID regimen), but they experienced a treatment delay in receiving PEP with no RIG. It is unfortunate that 6 people have died of rabies despite the completion of PEP. We have no information that revealed the exact cause of PEP failure in these cases. We, however, assume that this might be due to treatment delay or administering no RIG following exposed to suspected animal bites. While an estimated 10 million people receive rabies PEP every year after exposure to suspected animal bites, there are only sporadic reports of PEP failure exist. The various reasons for the probable or possible failure of rabies PEP include RIG not being used at all or incorrect administration, poor quality vaccine or RIG, the introduction of an exceptionally large viral load, virus injected directly into the nerve, unrecognized or unreported deviations from the WHO PEP protocol^48^. In Bhutan, 40% of rabies victims receive an incomplete vaccine course^47^. Approximately 33% of patients with dog bite cases in Thailand neglected to come for the last dose of the PEP series and failed to finish a complete series of PEP^49^. Experts have underscored the need for an improved PEP regimen requiring fewer hospital visits, helping to improve patient compliance in order to achieve PEP course completion and to minimize the victim’s burden of lost time and money^50,51^.

Stray dogs comprised the majority of exposures (90%), followed by stray cats (6%), jackals (3%), and mongooses (1%). A similar pattern was reported by other studies in Bangladesh and neighbouring countries^16,20,52^. We found, however, that cat, jackal and mongoose exposures were not properly addressed by the rabies victims in terms of seeking medical attention and PEP. This may be due to inadequate knowledge about the gravity of the disease and its host range. The vaccination status of the attacking animals (including dogs) was unknown in this study, indicating a higher number of free-roaming/stray dogs with no registration and vaccination records. Hence, along with continuing to scale up MDV, strategies to reduce stray dogs are essential; these measures have been documented to be a more advantageous, cost-effective, rational and ethical way to control rabies^53,54^.

The majority of the rabies patients died at home and only a fraction of them died in a health facility or hospital. The main underlying reason for this is that the socio-cultural practice in the country where the relatives of the rabies victims were not willing to stay in the hospital once they were informed about the grave prognosis of rabies. At that time, relatives urged the hospital management to discharge the patients from the IDH. This is the condition not only in Bangladesh but also in other developing countries, where taking a suspect/confirmed rabid patient to the hospital and bringing the dead body home is a burden for impoverished families^14^. Unfortunately, there are some countries where many rabies patients are turned away from hospitals and are only receiving terminal care from their families. Whenever possible, hospital care for patients with clinical rabies is advisable to reduce their suffering and ensure adequate, respectful palliative care^24^.

Our study had limitations. We have estimated the mortality rates based on the human rabies cases visited the public hospital facilities (NRPCC/DRPCCs). However, we are aware that we might have missed some cases, especially those who attended private hospital facilities or sought treatment from traditional healers and never visited the public hospital facilities. Thus the proportion we are reporting might be a fraction of total cases observed in Bangladesh. However, because of improvement in education and awareness, we believe only a few rabies deaths were missed by the government mainstream data-collecting centres. Identifying the occurrence of paralytic rabies was not possible, and it is not practical to diagnose this condition on clinical grounds. The present investigation was a retrospective study and there may have been recall bias as historical recollections depended almost entirely on the patients’ relatives. We, however, tried to mitigate this bias by calling some of the patients’ relatives directly. Despite these drawbacks, we believe that our investigation has revealed helpful information about rabies in Bangladesh and could be utilized in underdeveloped countries to control rabies.

## Conclusion

Our study showed that mass dog vaccination (MDV) is one of the most important components of controlling human rabies in Bangladesh. Also, these data clearly spelt out that most deaths had occurred as a result of consultation with the traditional healers instead of seeking post-exposure prophylaxis (PEP) from the hospitals and also due to the incomplete treatment course. Poor health-seeking behaviour of the rabies victims indicates the necessity to improve such behaviour through advocacy, communication, and social mobilization. It is necessary to address the role of traditional healers through an awareness education programme with respect to the treatment of dog bites and rabies and discouraging animal bite victims from visiting them. Ensuring the affordability and availability of rabies PEP in all areas of Bangladesh, especially in local public hospitals, is also important. Proper vaccine delivery needs sufficient personnel training to ensure correct storage, reconstitution, and injection. Sharing local epidemiological knowledge of rabies in animals may assist clinicians in making the right choice in treating rabies with PEP. We recommend conducting a humane method of dog population management programme along with the promotion of dog ownership and the continuation of scaling up MDV throughout the country to eliminate dog-mediated human rabies in Bangladesh. In addition, establishing a laboratory for rabies diagnosis and introducing an active surveillance system is necessary to monitor and evaluate emerging patterns and trends of the disease in Bangladesh. Strengthening and encouraging multi-sectoral involvement through the One Health approach is necessary for the sustainability of the rabies elimination programme in Bangladesh.

## Supplementary information

Supplementary Information.

## Data Availability

The datasets generated during and/or analysed during the current study are available from the corresponding author on reasonable request.
