# Supplementary material for: Trends and clinico-epidemiological features of human rabies cases in Bangladesh 2006–2018
Source: Sci Rep. 2020 Feb 12;10:2410. doi: 10.1038/s41598-020-59109-w (PMC7016137; doi:10.1038/s41598-020-59109-w)
Supplement: Supplementary file 1 — Supplementary Information. [file 41598_2020_59109_MOESM1_ESM.pdf]

Supplementary documents:

Trends and clinico-epidemiological features of human rabies cases in Bangladesh 2006-2018

Sumon Ghosh[a*], Md. Sohel Rana[a,i], Md. Kamrul Islam[a], Sukanta Chowdhury[b], Najmul Haider[c,j], Mohammad Abdullah Heel Kafi[b], Sayed Mohammed Ullah[a], Md. Rashed Ali Shah[a], Afsana Akter Jahan[a,d], Hasan Sayedul Mursalin[a], Aung Swi Prue Marma[a], S M Emran Ali[e], Shohrab Hossain[f], Rajub Bhowmik[g], Nitish C Debnath[h], Abul Khair Mohammad Shamsuzzaman[a], Be-Nazir Ahmed[a], Umme Ruman Siddiqi[a] and Sanya Tahmina Jhora[a]

[a] Disease Control Unit, Communicable Disease Control, Directorate General of Health Services, Ministry of Health and Family Welfare, Bangladesh
[b] International Centre for Diarrhoeal Disease Research, Bangladesh (icddr,b), Dhaka, Bangladesh
[c] Technical University of Denmark, Section for Epidemiology, National Veterinary Institutes, Copenhagen, Denmark
[d] Faculty of Veterinary and Animal Sciences, Gono University, Savar, Dhaka, Bangladesh
[e] Infectious Disease Hospital, Directorate General of Health Services, Ministry of Health and Family Welfare, Bangladesh
[f] Tongi Municipality, Tongi, Bangladesh
[g] John Jay College of the City University of New York, 445 W 59th St, New York-10019
[h] Food and Agriculture Organization of the United Nation, Dhaka, Bangladesh
[i] Department of Livestock Services, Ministry of Fisheries and Livestock, Bangladesh
[j] Department of Pathobiology and Population Sciences, Royal Veterinary College, University of London, UK

*Corresponding author. 3014 (2nd floor), IPH Building, icddr,b, 68 Shaheed Tajuddin Ahmed Sarani, Mohakhali, Dhaka 1212, Bangladesh.

E-mail address: sumon.ghoshbd@gmail.com (S. Ghosh)

Supplementary figures and tables

Table S1: Characteristics of animals to which the deceased rabies victims had been exposed, as reported at the IDH, Dhaka, Bangladesh, 2011-2015

Variables/categories	N=422<br>n(%)
Source of exposure	
Dog	380 (90)
Cat	24 (6)
Jackal	12 (3)
Mongoose	6 (1)
Types of animals/dogs	
Community dog	4 (1)
Pet dog	10 (2)
Stray dog	390 (93)
Wild animal	18 (4)
Cause of bites	
Provoked	110 (26)
Unprovoked	312 (74)
Fate of the animals	
Dead	41 (10)
Killed	72 (17)
Unknown	309 (73)
Vaccination status of the animals	
Non-vaccinated	10 (2)
Unknown	412 (98)

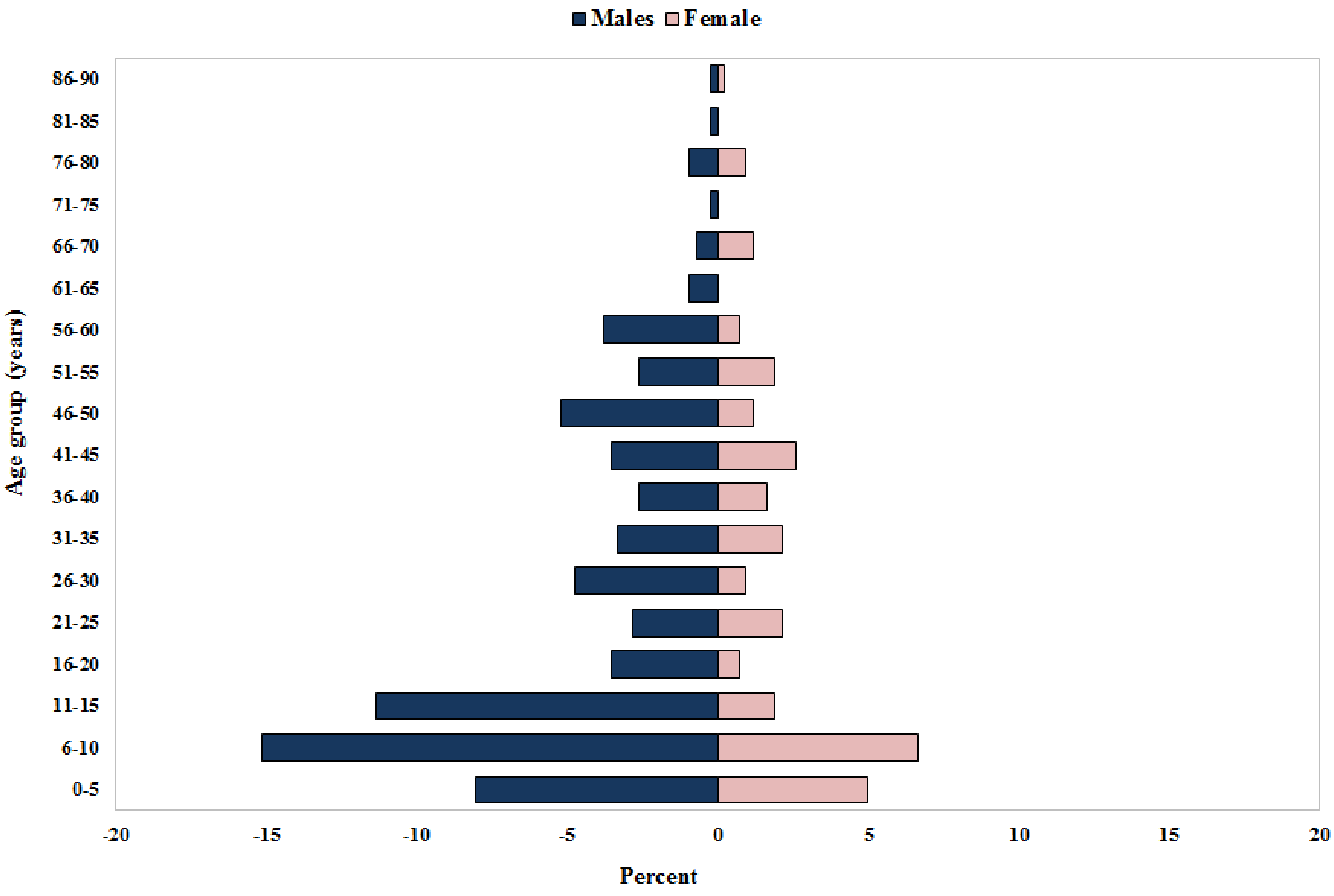

Figure S1. Distribution of human rabies cases based on gender and age group reported by the IDH, Dhaka, Bangladesh, 2011-2015. The cumulative proportion of all male patients in different age group sums up as 100% and the same as for all the female patients.

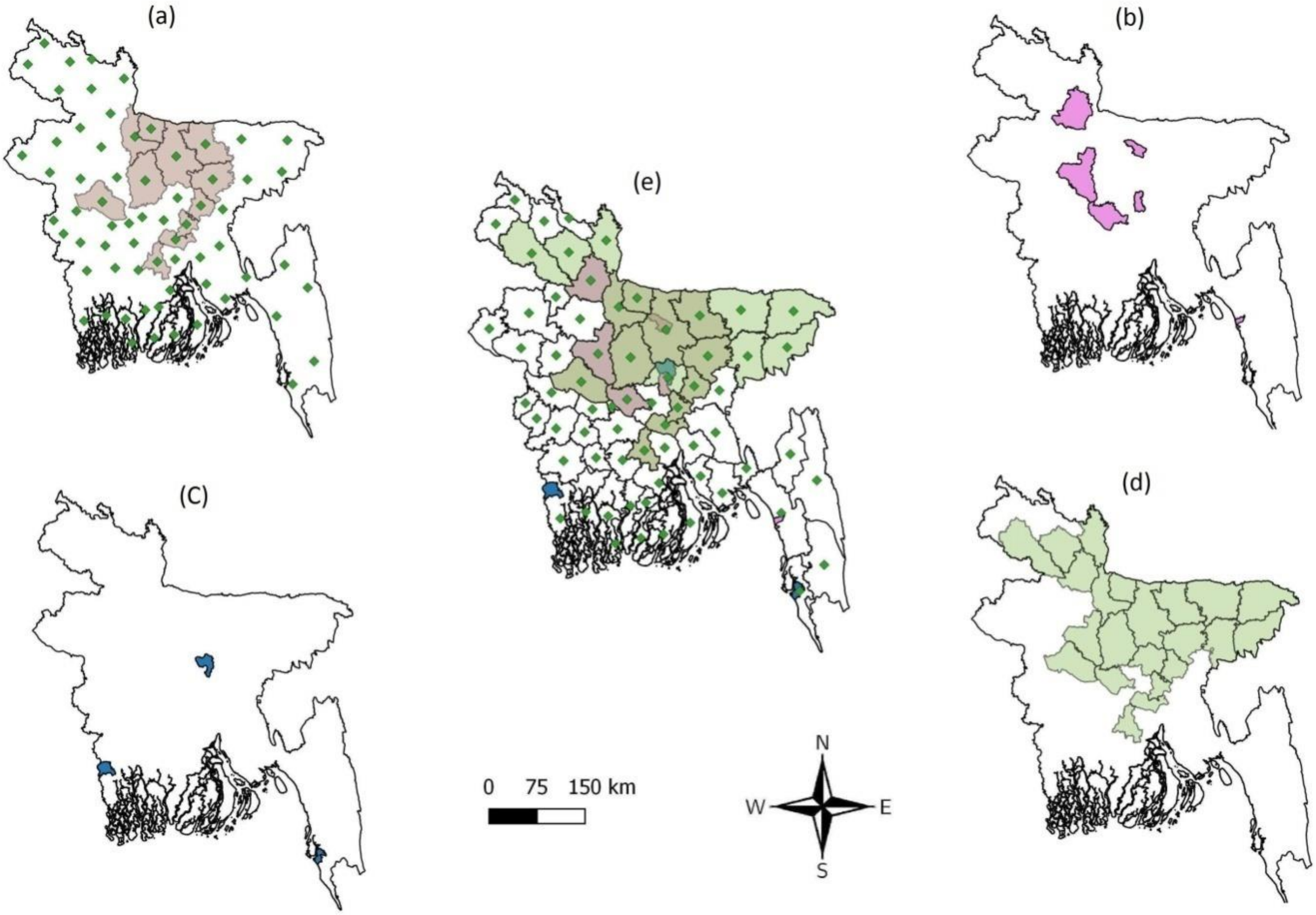

Figure S2. Scaling up MDV in Bangladesh (2011-2018). (a) Area covered with a single round of MDV [the green diamond (◆) indicates the district municipality], (b) Area covered with two rounds of MDV, (c) Area covered with three rounds of MDV, (d) All areas of the district (both municipalities and villages) covered with MDV (e) MDV in Bangladesh at a glance

Table S2: Estimated dog population and dog vaccination with vaccination coverage (%) in Bangladesh, 2011-2018

Year	Estimated dog population	Total number of dog vaccinated	Vaccination Coverage (%)
2011	4500	3285	73
2012	54980	51474	84
2013	18261	16089	87
2014	31334	26318	85
2015	180027	97711	77
2016	84420	70303	83
2017	50059	40420	81
2018	448184	365316	82

Table S3: Monthly dog vaccination with vaccine coverage in different districts, sub-districts and municipalities of Bangladesh, 2011-2018

District	Year	Month	Number of dog vaccinated	% Vaccination coverage	District	Year	Month	Number of dog vaccinated	% Vaccination coverage
Cox's Bazar	2011	November	3285	73	Chandpur	2013	May	1105	86
Dhaka	2012	March	214	71	Rangamati	2013	May	1029	86
Rangpur	2012	April	1907	86	Chittagong	2014	February	5688	77
Gaibandha	2012	April	538	76	Comilla	2014	March	2158	88
Kurigram	2012	April	845	76	Sylhet	2014	April	2175	93
Lalmonirhat	2012	April	733	82	Satkhira	2014	April	1374	94
Nilphamari	2012	April	1353	81	Cox's Bazar	2014	April	4030	79
Dinajpur	2012	April	1601	78	Khulna	2014	April	3847	88
Panchagarh	2012	April	815	40	Jhalokati	2014	May	484	80
Thakurgaon	2012	April	950	82	Barisal	2014	May	3463	87
Naogaon	2012	June	1485	91	Pirojpur	2014	May	911	76
Chapai Nawabganj	2012	June	1054	81	Barguna	2014	May	472	90
Bogra	2012	June	2591	86	Patuakhali	2014	May	588	81
Joypurhat	2012	June	621	75	Bhola	2014	May	826	84
Natore	2012	June	954	75	Moulvibazar	2014	May	302	80
Sirajganj	2012	June	1446	87	Gaibandha	2015	January	22484	75
Pabna	2012	July	1084	96	Sirajganj	2015	February	24864	74
Rajshahi	2012	July	4397	85	Pabna	2015	March	20147	76
Hobiganj	2012	July	524	91	Gazipur	2015	April	29975	77
Moulvibazar	2012	July	429	94	Manikgonj	2015	May	20777	77
Sunamganj	2012	July	560	89	Madaripur	2015	June	17022	85
Narayanganj	2012	August	3323	78	Dhaka	2016	January	7970	85
Gazipur	2012	August	5562	85	Chittagong	2016	February	3805	89
Narshingdi	2012	August	1250	94	Kishoreganj	2016	March	17829	82
Shariatpur	2012	August	976	92	Magura	2016	April	4730	79
Rajbari	2012	August	313	90	Naogaon	2016	May	7901	81
Madaripur	2012	August	625	81	Mymensingh	2016	June	6256	79
Gopalganj	2012	August	527	86	Gopalganj	2016	July	12124	84
Foridpur	2012	September	1059	84	Nilphamari	2016	August	9688	81
Kishoreganj	2012	September	1341	90	Gaibandha	2017	June	21644	80
Mymensingh	2012	September	1510	85	Gazipur	2017	August	12818	81
Netrokona	2012	September	1173	86	Nilphamari	2017	September	5958	81
Sherpur	2012	September	635	84	Manikgonj	2018	March	19660	78
Jamalpur	2012	September	1024	91	Narshingdi	2018	March	18924	84
Manikgonj	2012	September	1097	87	Narayanganj	2018	April	12176	75
Munsiganj	2012	September	1139	89	Gazipur	2018	April	12478	79
Kushtia	2013	February	913	86	Sirajganj	2018	April	25339	77
Jessore	2013	February	1335	88	Munsiganj	2018	April	15990	79
Chuadanga	2013	February	794	84	Jamalpur	2018	April	15035	78
Narail	2013	February	725	81	Sherpur	2018	April	12277	78
Jhenaidah	2013	February	927	86	Mymensingh	2018	May	43298	80
Magura	2013	February	975	92	Kishoreganj	2018	May	26002	83
Bagerhat	2013	February	1023	91	Netrokona	2018	June	23587	81

Satkhira	2013	February	1108	83	Tangail	2018	June	35409	79
Meherpur	2013	February	1026	88	Narayanganj	2018	August	5485	92
Noakhali	2013	April	916	86	Comilla	2018	August	817	83
B.Bariya	2013	April	805	91	Chittagong	2018	September	8470	89
Khagrachari	2013	April	1174	92	Gazipur	2018	September	3840	86
Lakshmipur	2013	April	565	74	Sylhet	2018	October	26708	83
Feni	2013	April	1154	83	Sunamganj	2018	October	22044	86
Bandarban	2013	April	515	80	Moulvibazar	2018	November	18310	88
					Hobiganj	2018	November	17895	85
					Dhaka	2018	November	1572	88

Figure S3: Monthly human rabies and dog rabies vaccination in Bangladesh, 2006-2018

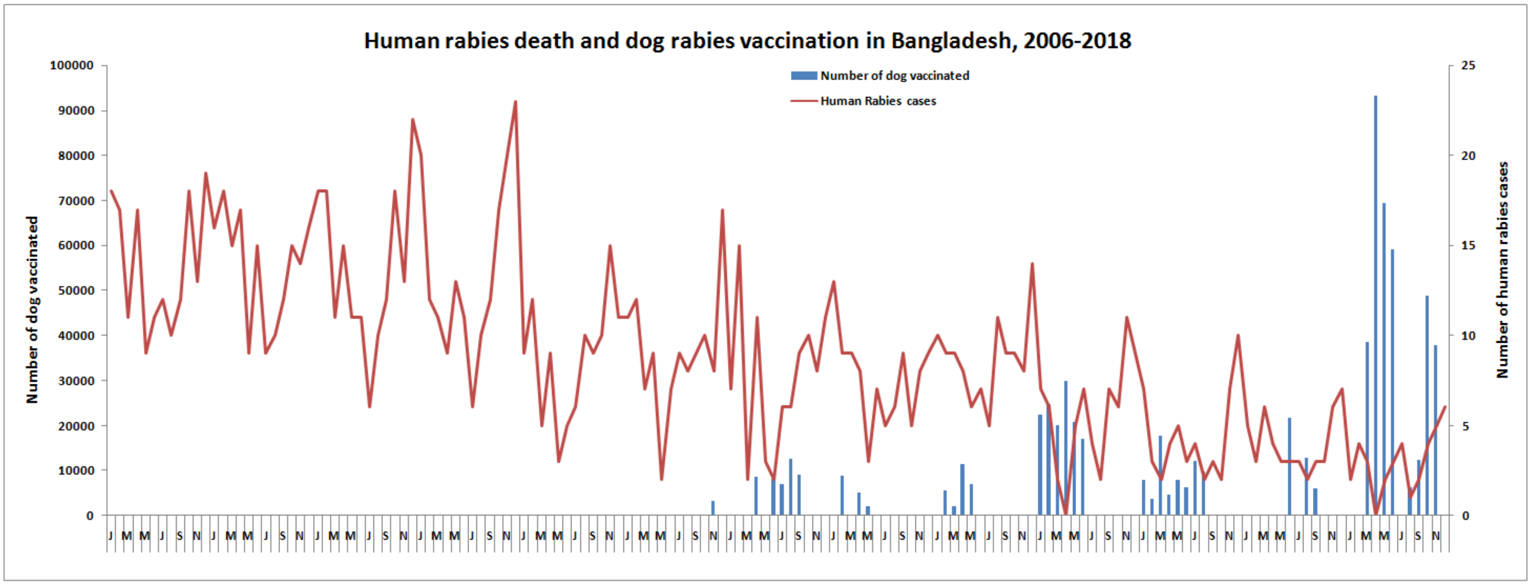

Table S4: Animal bite patients managed and PEP utilization at NRPCC and DRPCC of Bangladesh, 2011-2015

	**No of animal bite cases managed at NRPCC & DRPCC**					
**Year**	**Category I#**	**Category II##**	**Category III###**	**Total**	**Total number of animal bite patient who received Anti Rabies Vaccine (ARV)**	**Total number of animal bite patient who received both ARV & RIG**
2011	.	.	.	45536	115	
2012	6164	86296	36984	129444	5909	112
2013	16504	160324	75447	252275	190456	8176
2014	17243	163791	73587	254621	96558	3770
2015	17083	165287	78132	260502	53652	3280
**Total**	56994	575698	264150	942378	346690	15338
**Average**	14248	143924	66037	188476	69338	3834

#Category I—touching or feeding an animal or licks on intact skin: no exposure; PEP not indicated;
##Category II—nibbling of uncovered skin, minor scratches or abrasions without bleeding: exposure; PEP indicated with vaccine; to be treated as category III if exposure was to a bat;
###Category III—single or multiple transdermal bites or scratches, contamination of mucous membranes with saliva from licks, licks on broken skin, exposure due to direct contact with bats: severe exposure; PEP indicated with vaccine and RIG.
[Rabies vaccines WHO position paper–April 2018 Wkly Epidemiol Rec, 93 (2018), pp. 201-220]
